# *In Utero* Exposure to Dioxins and Polychlorinated Biphenyls and Its Relations to Thyroid Function and Growth Hormone in Newborns

**DOI:** 10.1289/ehp.7994

**Published:** 2005-06-27

**Authors:** Shu-Li Wang, Pen-Hua Su, Shiang-Bin Jong, Yueliang L. Guo, Wei-Ling Chou, Olaf Päpke

**Affiliations:** 1 Division of Environmental Health and Occupational Medicine, National Health Research Institutes, and Graduate Institute of Occupational Safety and Health, Kaohsiung Medical University, Kaohsiung, Taiwan; 2 Institute of Medicine, Chung Shan Medical University, and Department of Pediatrics, Chung Shan Medical University Hospital, Taichung, Taiwan; 3 Department of Nuclear Medicine, Kaohsiung Medical University Hospital, Kaohsiung, Taiwan; 4 Department of Occupational and Environmental Health, College of Medicine, National Cheng Kung University, Tainan, Taiwan; 5 ERGO Research Laboratory, Hamburg, Germany

**Keywords:** dioxins, infant, placenta, prenatal exposure delayed effects, thyroid hormones

## Abstract

The aim of this study is to examine the association between transplacental exposure to dioxins/polychlorinated biphenyls (PCBs) and thyroid and growth hormones in newborns. We recruited 118 pregnant women, between 25 and 34 years of age, at the obstetric clinic. Personal data collected included reproductive and medical histories and physical factors. Clinicians gathered placental and umbilical cord serum upon delivery and carefully scored the 118 newborns, making both structural and functional assessments. We analyzed placentas for 17 polychlorinated dibenzo-*p*-dioxins and dibenzofurans and 12 dioxin-like PCB congeners with the World Health Organization–defined toxic equivalent factors, and six indicator PCBs by high-resolution gas chromatography and high-resolution mass spectrometry. We analyzed thyroid and growth hormones from cord serum using radioimmunoassay. Insulin-like growth factor (IGF)-1, IGF-binding globulin-3, and thyroxine × yroid-stimulating hormone (T_4_ × TSH) were significantly associated with increased placental weight and Quetelet index (in kilograms per square meter; correlation coefficient *r* = 0.2–0.3; *p* < 0.05). Multivariate analyses showed independently and significantly decreased free T_4_ (FT_4_) × TSH with increasing non-*ortho* PCBs (*r* = −0.2; *p* < 0.05). We suggest that significant FT_4_ feedback alterations to the hypothalamus result from *in utero* exposure to non-*ortho* PCBs. Considering the vast existence of bioaccumulated dioxins and PCBs and the resultant body burden in modern society, we suggest routine screening of both thyroid hormone levels and thyroid function in newborns.

Polychlorinated dibenzo-*p*-dioxins and dibenzo-furans (PCDD/PCDFs), known together as dioxins, and polychlorinated biphenyls (PCBs) are ubiquitous, persistent organic pollutants that act as endocrine disruptors in eco- and bio-systems. Various industries, the combustion of solid wastes, and forest fires generate these xenobiotic pollutants. Human milk surveys have established a high body burden for these substances in long-industrialized countries, such as those in Europe (Schecter and Piskac 2001). These contaminants are highly lipophilic and are potentially bioamplified through the food chain. Humans accumulate such compounds mainly from foods of animal origin, including fish (Smith and Gangolli 2002), meat, dairy products, and processed foods such as fish oil (Chen et al. 2003; Rideout and Teschke 2004). PCDD/PCDFs and PCBs have long half-lives of 7–10 years in humans. The body burden and related health effects are particularly concerning in countries with high population density, such as Taiwan, due potentially to the increased numbers of factories, solid waste incinerators (Tango et al. 2004), and the volume of imported foods from animal sources.

Our previous study established the transfer of PCDD/PCDFs and PCBs from mother to infant via the placenta (Wang et al. 2004) in the general population. These endocrine-disrupting effects upon thyroid function might significantly affect growth and development during fast growth stages of the central nervous system in the human fetus (Koopman-Esseboom et al. 1994; Pluim et al. 1992). These effects on fetal growth and development, particularly as related to the ectodermal layer (Guo et al. 2000; Jacobson and Jacobson 1996), are irreversible and can last for several years of life (Chen et al. 1992; Jacobson and Jacobson 1996; Patandin et al. 1999). Dental health might also be affected (Alaluusua et al. 1999; Wang et al. 2003); however, this mechanism awaits further investigation. Early hypo- or hyperthyroidism or thyroid receptor alterations can cause permanent behavioral, intellectual, and neurologic dysfunction, particularly during the second and third months of gestation (Utiger 1999). Human thyroid function is similar to that of other vertebrates, but the thyroid system is well developed and functioning at birth (Fisher and Brown 2000). Animal studies have shown that dioxins and PCBs might affect thyroid hormone secretion, transport, and/or action (Leatherland 1999). More recent studies have shown that low-dose 2,3,7,8-tetrachlorodibenzo-*p*-dioxin (TCDD) might cause rat thyroid morphologic and functional abnormalities through gestational and lactational exposure (Nishimura et al. 2003).

Different dioxin and PCB congeners might have different toxicities. The approach that uses toxic equivalent factors to summarize toxicity is subject to debate particularly for dioxin-like PCBs (Van den Berg et al. 1998), because the toxicity of dioxin-like PCBs in the presence of non-dioxin-like PCBs cannot be summarized simply by additive effects. The present study provides an opportunity to examine both single and summarized levels.

There have been inconsistent outcomes regarding thyroid function alterations resulting from dioxins and PCBs in humans with background exposure in both the Netherlands (Koopman-Esseboom et al. 1994; Pluim et al. 1993) and Japan (Matsuura et al. 2001; Nagayama et al. 1998). Such diverse thyroid hormone findings might result from using different matrices (Kimbrough and Krouskas 2001) for measuring exposure (i.e., breast milk or venous blood), various specimen collection time points, and/or studied isomers (dioxins, indicator PCBs) for different populations at unique sample sizes. The aim of this study is to examine the association between transplacental exposure to various dioxin and PCB congeners and thyroid hormone status in 118 mother–newborn pairs from the general Taiwanese population, taking into account growth hormones and birth outcomes.

## Materials and Methods

### Subjects.

This is part of a prospective study of dioxins/PCBs for the general population in central Taiwan. Subject recruitment was described in a previous study (Wang et al. 2004) with a detailed birth cohort (Chao et al. 2004). In summary, we invited all pregnant women visiting the local medical center between December 2000 and November 2001 to participate in this study. We interviewed 430 subjects at the obstetric clinic, collecting personal data that included reproductive and medical histories, and physical factors. The subjects were all between 25 and 34 years of age with a single pregnancy and without known complications, cigarette smoking, or alcohol consumption during the pregnancy. Clinicians gathered placental and umbilical cord blood upon neonate delivery. One well-trained nurse carefully scored all newborns, including both structural and functional assessments, which a physician verified.

The Human Ethical Committee of the National Health Research Institutes in Taiwan reviewed and approved the study protocol. This study followed the ethical standards formulated from the Helsinki Declarations of 1964 and revised in 2000 (World Medical Association 2000). Each of the participants provided informed consent after receiving a detailed explanation of the study and its potential consequences.

### Specimen and data collections.

Immediately after collection on the ward, technicians divided each placenta into two equally symmetrical parts, taking half for the study sample. Technicians then sliced each sample into pieces, storing 100 g of each sample in Teflon-lined cap glass bottles for future dioxin and PCB measurements, and then shipped the frozen placental sections to O. Päpke in ERGO Research Laboratory (Hamburg, Germany), which is certified by the World Health Organization (WHO), for analysis. This laboratory regularly and successfully participates in international interlaboratory studies, including PCDD/PCDFs in human milk, beef, and fish liver (Päpke 1998).

Analyses of PCDD/PCDFs and PCBs were performed according to a previously published method (Päpke et al. 1994). Briefly, we extracted 100-g placenta samples with *n*-pentane after adding an internal standard, such as ^13^C_12_-PCDD/PCDF or ^13^C_12_-PCB. We determined the lipid content of the samples using a gravimetric method before cleanup in a multicolumn system. The congeners, with the WHO-defined toxic equivalent factors (Van den Berg et al. 1998) of seventeen 2,3,7,8-substituted PCDD/PCDFs, 12 dioxin-like PCBs including non-*ortho* and mono-*ortho* PCBs, and six indicator PCBs [International Union for Pure and Applied Chemistry (IUPAC) congeners 28, 52, 101, 138, 153, and 180] were analyzed by gas chromatography (Hewlett Packard GC 5890 Series II; GMI Inc., Ramsey, MN, USA) with high-resolution mass spectrometry (VG-AutoSpec, Manchester, UK). We purchased authentic standards of native dioxin-like PCBs and PCDD/PCDFs from AkkuStandard Inc. (New Haven, CT, USA). We obtained indicator PCB standards from LGC Promochem (Wesel, Germany). We then measured two isotope masses for each component. We used internal/external standard mixtures via the isotope dilution method for quantification.

In our study we examined 118 newborns with complete data including dioxin/PCB levels in the placenta and thyroid hormone status via the cord serum. We noted increased thyroid hormone levels during the first few hours of life due to the expected postparturition surge of thyroid-stimulating hormone (TSH). Thyroid hormone concentration measurements in the cord blood were similar to those measured in the venous blood at 24–72 hr or 2 weeks of age (Mashang and Thornton 1999). We collected cord blood upon umbilical cord ligation, which we completed within 1 min of childbirth; thus, we used cord levels as indices of thyroid function without phlebotomizing the infants. The decreased number of subjects reported is a result of the economic dioxin analyses necessary to achieve a statistical power sufficient to generate a valid conclusion. Thyroid and related growth hormones were measured using radio-immunoassay methods, including triiodothyronine (T_3_), thyroxine (T_4_), thyroid-stimulating hormone (TSH; thyrotropin), free T_4_ (FT_4_), T_3_ uptake, thyroid-binding globulin (TBG), insulin-like growth factor (IGF)-1, and IGF-binding globulin-3 (BP3). We carried out blind duplicates for every 10 samples. We purchased T_3_, T_4_, TSH, IGF-1, and BP3 commercialized kits from Daiichi Radioisotope Laboratory (Tokyo, Japan). FT_4_ kits were from CIS Bio International US Inc. (Bedford, MA, USA); T_3_ uptake kits were from Diagnostic Products Corporation (Los Angeles, CA, USA); TBG kits were from CIS BIO International (Marcoule, France).

### Statistical methods.

**Figure 1:**
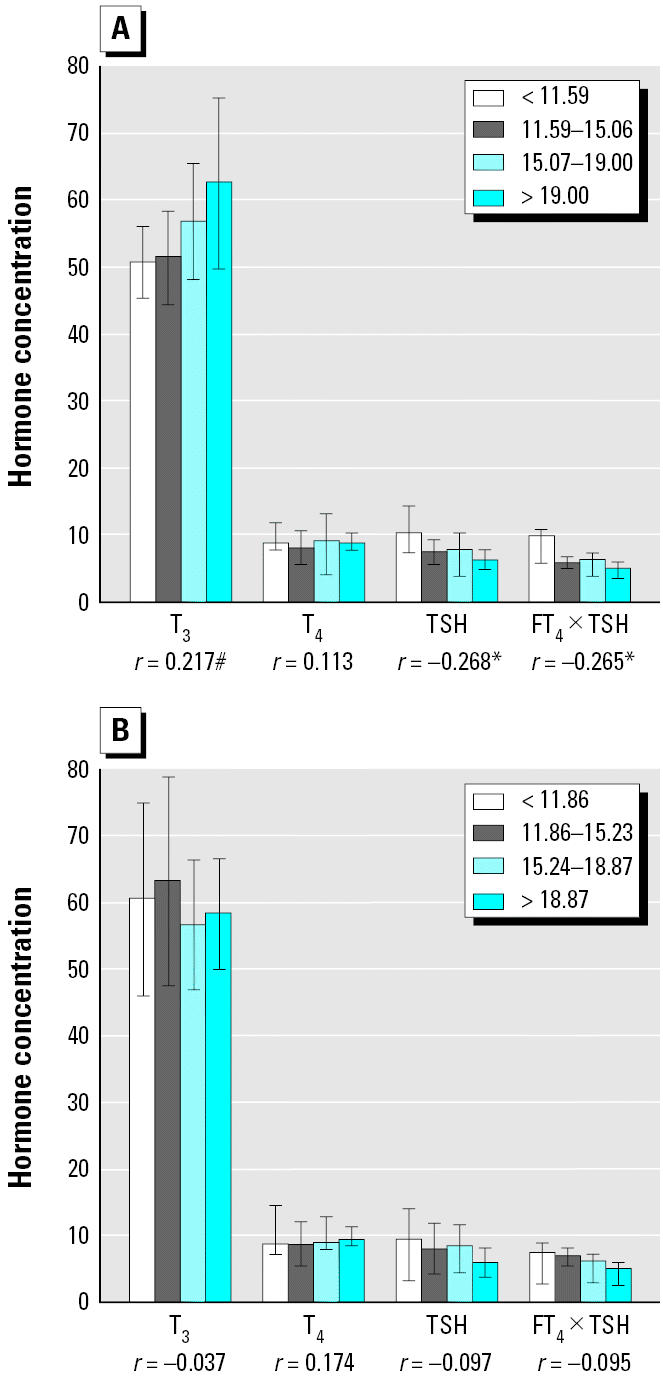
Concentrations of T_3_ (ng/dL), T_4_ (μg/dL), TSH (μU/mL), and FT_4_ × TSH (ng/dL × μU/mL) in cord serum according to quartiles of total dioxins and PCBs (pg-TEq/g lipid) in placenta: (*A*) females; (*B*) males. *p < 0.05; #*p* < 0.1.

**Figure 2:**
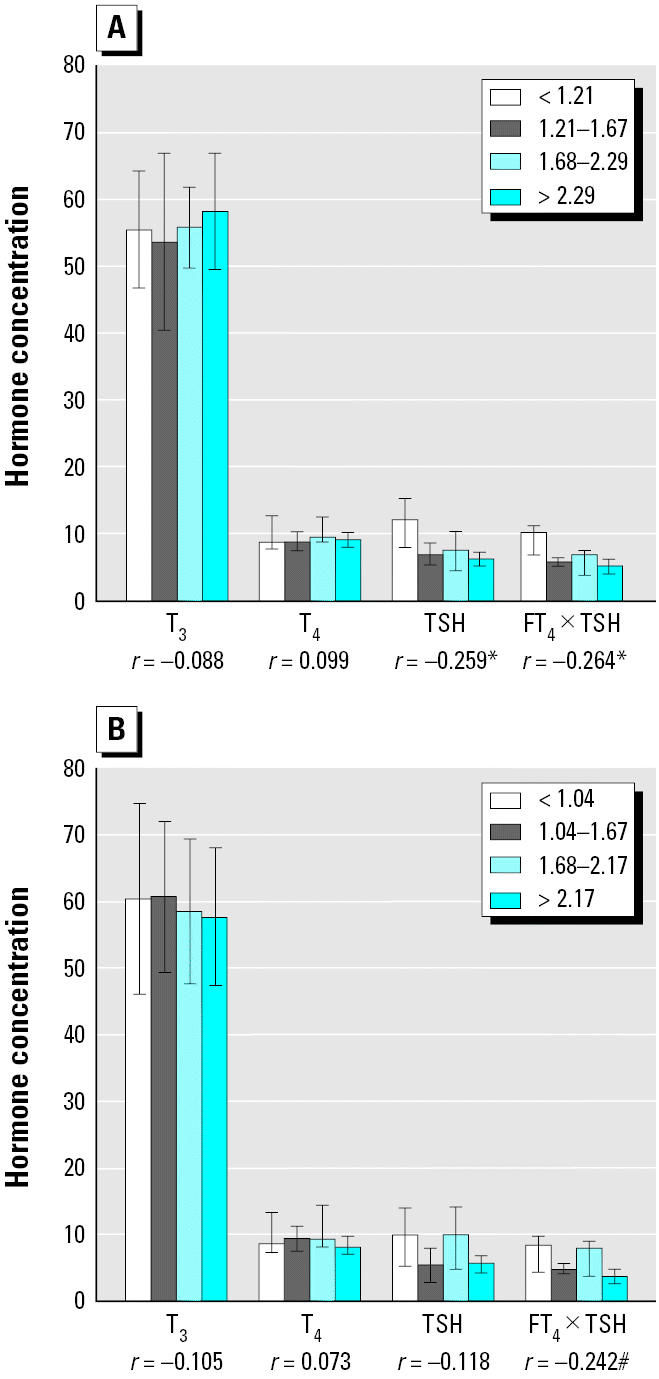
Concentrations of T_3_ (ng/dL), T_4_ (μg/dL), TSH (μU/mL), and FT_4_ × TSH (ng/dL × μU/mL) in cord serum according to quartiles of non-*ortho* PCBs (pg-TEq/g lipid) in placenta: (*A*) females; (*B*) males. *p < 0.05; #*p* < 0.1.

We verified the data distribution of each continuous variable for normality and generally noted data skewing slightly to the right. When data distribution was significantly beyond the normal distribution range, we used the Mann-Whitney *U*-test for determining differences (i.e., hormone levels) between high- and low-exposure groups; otherwise, we performed the Student *t*-test. We used the chi-square test for categorical variables. We calculated the multiplied concentrations of FT_4_ × TSH based on the normal hypothalamic–pituitary axis in which decreased T_4_ feeds back to the hypothalamus and thereby stimulates the anterior pituitary to secret TSH (Scanlon and Hall 1998). We also calculated the concentration ratio of T_4_:TBG because TBG is the main T_4_ carrier protein, and generally there is positive association between the concentrations of T_4_ and TBG (Porterfield 2001). We used Spearman correlation analyses to evaluate the association between PCDD/PCDF and PCB levels and hormone levels. For the present report, we confirmed the results using Pearson correlation analyses after log transformation. We used a general linear model to examine the relationship of the hormone log values to the dioxin/PCB exposure values. To increase statistical power, we combined the results of male and female infants when their patterns were the same. We carried out multivariate linear regression analyses to adjust for age and other appropriate variables. We used the Statistical Package for Social Science (version 10.0; SPSS, Chicago, IL, USA) to execute all statistical analyses. For data presentation, we used international units (IU), except for Figures 1 and 2 in which IU was not used to illustrate different hormone levels in single figures.

## Results

The newborns were generally healthy with a mean (± SD) body weight of 3,229 ± 371 and Apgar scores at 1 and 5 min of 8.4 and 9.7, respectively. The mothers had a mean (± SD) age of 29 ± 4 years (Table 1). Male infants tended to have greater birth weights and head girths (*p* < 0.1) than did female infants. Approximately half of the infants were first parity, with an even distribution of males and females. Higher dioxin/PCB exposure groups correlated significantly with increased maternal age and longer infant length. Higher parity might confound the length relationship, although this relationship did not persist with multivariate analysis. One- and 5-min Apgar scores were both slightly lower in the high-exposure group, but this was without statistical significance (*p* = 0.19, 0.35, respectively). Table 2 demonstrates significantly increased concentrations of T_3_, TBG, and BP3 and decreased TSH in female infants, whereas in males, decreased TSH values were not statistically significant. The 95% confidence intervals (CIs) for T_3_, T_4_, TSH, and FT_4_ were 54.3–60.6 (ng/dL), 8.4–9.1 (μg/dL), 6.8–8.9 (μU/mL), and 0.77–0.85 (ng/dL), respectively. The concentrations in the present study were within the normal range for cord blood (Mashang and Thornton 1999). We excluded one subject who was seen in follow-up for thyroid dysfunction and dropped out of the study from the present report.

Maternal age was significantly associated with decreased gestational age and Apgar scores (Table 3). Further analyses showed that maternal age correlated with increased parity and was associated with increased infant weight and length. Therefore, the greater the parity, the shorter the gestational age for an infant with a normal-range weight. IGF-1 and BP3 concentrations in cord serum were significantly and positively associated with greater placental weight, birth weight, head girth, and Quetelet index (QI) (correlation coefficient, 0.2–0.3). FT_4_ × TSH and T_4_ × TSH were both significantly and positively associated with placental weight and QI (*r* = 0.2–0.3). Maternal thyroid hormones at the third trimester of pregnancy did not correlate with birth outcomes; therefore, these are not included in the present report.

We examined whether the key growth- and development-related hormones in cord blood were associated with dioxin/PCB body burden after adjusting for maternal age (Table 4). We found significant correlations mainly for female infants. First, increased T_3_ levels were associated with increased PCDFs. Second, decreased TSH levels were associated with increased PCDDs and dioxin-like PCBs. Third, increased TBG levels were associated with increased PCDD/PCDFs. Fourth, decreased FT_4_ × TSH levels were associated with increased non-*ortho* PCBs. Fifth, decreased T_4_:TBG and T_3_ uptake:T_3_ levels were associated with increased PCDD/PCDFs. The concentrations of PCB-138, -153, and -180 were not significantly associated with key hormone levels and related only somewhat to T_4_ and FT_4_ levels in females.

Table 5 shows the results of multivariate analyses for significant relationships between the hormone and dioxin/PCB levels after adjusting for maternal age and other dioxin/PCB isomers. We combined PCDDs and PCDFs with the same relationship pattern to increase statistical power. We noted the continuation of a significant and positive association between T_4_ concentrations with levels of PCDD/PCDFs. We also noted a significant relationship between TBG levels and increased PCDF levels. FT_4_ × TSH levels were negatively associated with non-*ortho* PCB toxic equivalents (TEq; *r* = −0.25) and positively related to concentrations of PCB-138, -153, and -180 (*r* = 0.02; data not shown) independent of age and other congeners. We noted a negative relationship between FT4 levels and non-*ortho* PCB levels. In addition, maternal age was negatively associated with T_4_, TBG, and FT_4_ × TSH. Mainly because of low levels and values below the detection limit, mono-*ortho* PCBs have not demonstrated a significant association with hormone levels.

**Figure 3:**
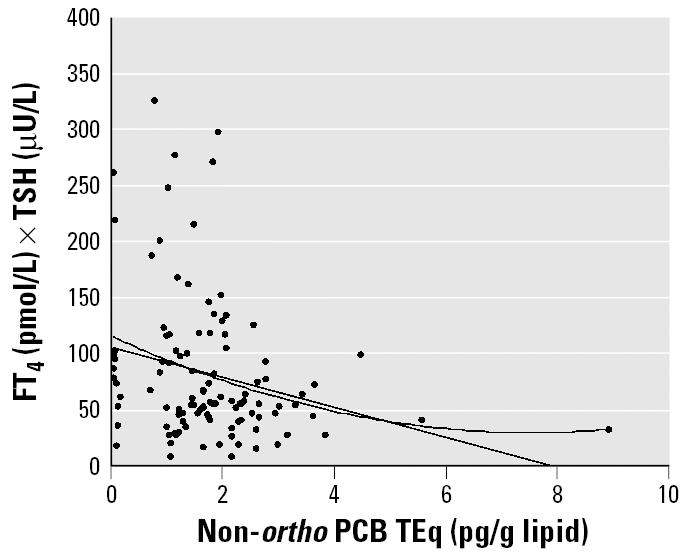
Decreased FT_4_ × TSH levels according to concentrations of non-*ortho* PCBs. By general linear model: FT_4_ × TSH = 8.32–1.05 × (non-*ortho* PCB); *R* = 0.245, *p* = 0.009. By quadratic model: FT_4_ × TSH = 9.06–1.77 × (non-*ortho* PCB) + 0.116 × (non-*ortho* PCB)^2^; *R* = 0.261, *p* = 0.022.

We also noted increased T_3_ and T_4_ concentrations, as well as decreased TSH and FT_4_ × TSH, with increasing total dioxins and PCB TEq levels, the latter two statistically significant for female infants (Figure 1A). For male infants, similar patterns were not statistically significant (Figure 1B). Non-*ortho* PCBs were associated with decreased FT_4_ × TSH for both sexes after examining various congener groups (Figure 2). Each subject’s level was plotted for non-*ortho* PCBs against FT_4_ × TSH (Figure 3). Both linear and nonlinear models demonstrated a significant negative association. The 95% CIs for values of FT_4_ × TSH and T_4_ × TSH were 5.5–7.4 ng/dL × μU/mL and 58.3–78.5 μg/dL × μU/mL, respectively.

## Discussion

This is the first study demonstrating the *in utero* effects, as measured in the placenta, of dioxins and PCBs on thyroid function, considering various growth factors in newborn infants from the general population. Thyroid function is crucial for infant growth and development (Polk and Fisher 1998), and previous studies of 38 healthy, term infants have shown borderline significance for increased T_4_ and TBG concentrations in upper-median dioxin-exposed groups as measured in breast milk (Pluim et al. 1992, 1993). Here, we demonstrate consistently increased T_3_, T_4_, and TBG levels in cord blood correlating to upper-median exposure groups for female but not male infants. The increases in T_4_ and TBG levels seen with increasing dioxins persisted even after adjusting for maternal age and PCB exposure.

We further demonstrated decreased FT_4_ × TSH levels with increasing non-*ortho* PCBs, which remained significant even after adjusting for other dioxin and PCB congeners. This phenomenon was most noteworthy in female infants. FT_4_ × TSH levels could be predicted with significance by using a simple linear regression model with non-*ortho* PCB TEq levels as predictors. FT_4_ is the major hormone affecting hypothalamus-stimulating TSH (Scanlon and Hall 1998). FT_4_ feedback might not be effective enough to stimulate the hypothalamus to secrete TSH, resulting in decreasing TSH levels. We also demonstrated that TSH and FT_4_ × TSH are significantly and positively associated with placental weight and QI, signifying the general growth impact from the two hormones. We recommend routine thyroid function screening in newborns as a protective measure in the face of ubiquitous environmental endocrine disruptors, particularly for bioaccumulated and transplacental dioxins and the PCB body burden. Such screening should include both individual thyroid hormone levels (i.e., TSH and/or T_4_) and relationship values (i.e., FT_4_ × TSH) for functional evaluations. We did not emphasize the significant reduction of T_3_ and T_4_ concentrations with increasing non-*ortho* PCBs because of the positive association between the hormone levels and dioxins, particularly for T_4_ and PCDDs, possibly due to the increased TBG concentration with increasing PCDDs. Various conger considerations are important because of the multiple exposures that humans receive.

Regarding sex differences, we noted significant increases in BP3 in high-exposure female infants in the present study. Female infants exposed to slightly higher dioxin and PCB levels were without statistical significance, however, and these female infants generally had similar hormone levels to the male infants. In addition, female infants were slightly smaller and had higher bilirubin levels than did male infants. It was easier to differentiate hormone levels by upper- and lower-median dioxin/PCB levels in females, which indicated greater sensitively to endocrine disruptors in female compared with male neonates. Sex differences in thyroid hormones were also found in Michigan Lake fish eaters (Persky et al. 2001). Most experts consider dioxins antiestrogenic with context-dependent effects, which might explain the greater hormonal outcomes observed in females. Estrogen receptors in thyroid cells (Scanlon and Hall 1998) and greater responses of TSH to thyrotropin-releasing hormones in females might also contribute. Further investigation is necessary to elucidate the reasons behind these sex differences.

Prior research had different results than those of the present study. In a study of residents living in a highly industrialized region of the Netherlands (Koopman-Esseboom et al. 1994), dioxins and PCBs in breast milk collected at 14 days postpartum showed no association with T_4_, T_3_, FT_4_, and TSH concentrations in cord blood but showed significantly decreased T_4_ and increased TSH levels at 2 weeks and 3 months postpartum. Among these 78 mother–infant pairs collected from surrounding industrial areas of Rotterdam, TSH varied from 10.0 μIU/mL at birth to 2.3 μIU/mL at 2 weeks. Dioxins and PCBs in breast milk ranked high in Europe at 30.8 pg TEq/g fat compared with 15.1 in the present study; however, the method detection limit has been largely lowered because of technical improvements in the last 10 years. In addition, dioxin and PCB levels in breast milk might decrease with parity and duration of the feeding; therefore, it is difficult to make direct comparisons between the Dutch study and the present study. Nonetheless, both studies drew the same conclusions regarding altered thyroid hormone status as related to dioxins and PCB exposure, and both conclude that the data warrant further follow-up.

More recent studies of the general population in Japan showed no association between TSH and FT_4_ concentrations at 1 year with dioxins and PCBs in breast milk collected at 30 postpartum days (Matsuura et al. 2001). This indicated that breast-feeding might not change thyroid hormone levels significantly. As recommended by Rogan and Miller (1989), the ectodermal effects of *in utero* longer term dioxin and PCB exposure needs to be examined. Our study of dioxins and PCBs in 118 placentas demonstrated associated hormonal changes, while accounting for various growth factors and covariates. For instance, we noted higher dioxin and PCB body loads with increased maternal age, which results in increased transplacental and lactational exposure to fetus and infant, respectively. We suggest that researchers consider age when evaluating the effects of persistent thyroid hormone disruptors. In the present study, we found that maternal age was associated with increased dioxin levels, decreased gestational age, and decreased T_4_ and TSH concentrations, within 25–34 years of age. Maternal age adjustments in our study might explain why our outcomes differed from those of previous studies.

Animal studies have indicated that thyroid function changes according to gestational and lactational exposure to TCDD and PCBs (Fisher and Brown 2000; Nishimura et al. 2003; Rosiak et al. 1997). In the offspring of Holtzman rats sensitive to TCDD, serum T_4_ levels decreased on postnatal day 21 and increased by postnatal day 49 when exposed to a low-dose (either 200 or 800 ng/kg) at gestational day 15 (Nishimura et al. 2003). T_3_ increased at both postnatal day 21 and postnatal day 49, but this increase was less significant. Nishimura et al. (2003) concluded the induction of the uridine diphosphate-glucuronosyltransferase-1 gene in the liver increases T_4_ metabolism. In this animal study, hyperplasia of the thyroid gland might result from decreased T_4_ and sustained high TSH levels with a corresponding feedback system disorder. We observed greater T_3_ levels but did not observe increased TSH levels in female neonates in relation to increased PCDFs. Background exposure might affect humans differently than animal models. In addition, levels of TBG, the major T_4_ carrier protein in the peripheral blood (LaFranchi 2000), were significantly increased with dioxin exposure. TBG might be increased during pregnancy secondary to maternal estrogen effects (Mashang and Thornton 1999); therefore, researchers recommended further analyses of estrogens in cord blood. We found FT_4_ and T_3_ uptake were slightly and negatively associated with dioxins, indicating there might be fewer unoccupied binding sites on TBG. Additional studies to examine carrier protein changes using animal models would help further clarify regulation processes and help define the reference levels for pathologic diagnosis.

Although indicator PCBs have been associated with decreased T_4_ levels and sustained TSH stimulation in Sprague-Dawley pups (Ness et al. 1993), the present study found no major correlation between the various thyroid hormones and PCB-138, -153, and -180. The only correlation noted in this study was that of increased T_4_ levels with increasing concentrations of non-dioxin-like PCBs in female infants as determined by univariate analysis. Differences in exposure levels of endocrine disruptors might reveal different effects (Brouwer et al. 1999).

There are several methodologic concerns in studies of this nature. First, it is difficult to draw venous blood from infants who are < 3 months of age. Nonetheless, the pattern of hormonal changes remained consistent at 1 and 11 postnatal weeks according to observation of infants in the general population of the Netherlands (Pluim et al. 1993). Second, we did not include routine TSH regulatory monitoring results in the present report because venous concentrations varied with time points of the blood draws; thus, this was not suitable data for comparison. The pregnant women in our study did not have iodine insufficiency concerns to our understanding, probably because of the proximity to the sea and dietary habits, which include the intake of processed foods using seaweed containing iodine. For exposure measurements, human milk dioxin/PCB levels decreased substantially with the duration of breast-feeding. The percentage of all congener-specific analyses for PCDD/PCDF and PCB levels greater than the detection limit is 90% for placenta, compared with 75% for human milk and 43% for cord serum. Placental analyses might prove to be good indicators of *in utero* exposure for newborns.

## Conclusions

We found significantly decreased FT_4_ feedback to the hypothalamus resulting from *in utero* exposure to non-*ortho* PCBs in neonates from the general population. The mechanism of action of the compound treatment remains to be determined. We also recommend routine screening of both individual thyroid hormonal levels and thyroid function in newborns, considering the pervasive existence of bioaccumulated dioxins and PCBs and the resulting body burden in modern society. We recommend further studies that include antiestrogenic effects to examine the positive correlation between TBG and increased dioxins. It is worthwhile to follow the growth and development for those with altered thyroid status.

## Figures and Tables

**Table 1:** Birth outcomes by sex and median of dioxin and PCB TEq level in the newborn babies (mean ± SD).

	Sex (*n*)			Dioxin/PCB TEq^a^ (pg/g lipid)			
Factor (unit)	Female (*n* = 62)	Male (*n* = 57)	*p*-Value^b^	Low (< 15.1; *n* = 59)	High (≥ 15.1; *n* = 60)	*p*-Value^b^	Total factor (unit)
Dioxin/PCB TEq	16.38 ± 6.76	16.01 ± 5.44	0.75	11.59 ± 2.43	20.74 ± 5.24	< 0.001	16.20 (6.14)
Mother’s age	29.81 ± 4.60	28.65 ± 4.11	0.15	27.54 ± 4.03	30.93 ± 4.10	< 0.001	29.35 (4.04)
Placenta weight (g)	574.4 ± 150.4	571.7 ± 138.6	0.92	582.5 ± 165.5	563.7 ± 119.7	0.50	568.2 (122.5)
Placenta fat content (%)	0.75 ± 0.10	0.75 ± 0.13	0.98	0.75 ± 0.11	0.75 ± 0.11	0.94	0.77 (0.12)
Gestational age (weeks)	38.64 ± 1.33	39.15 ± 1.42	0.05	39.00 ± 1.31	38.76 ± 1.47	0.35	38.80 (1.37)
Baby birth weight (g)	3,051 ± 411.7	3,181 ± 416.3	0.09	3,062 ± 416.6	3,163 ± 415.3	0.19	3,229 (371.1)
Baby birth length (cm)	51.09 ± 2.27	51.66 ± 2.46	0.19	50.90 ± 2.34	51.82 ± 2.33	0.03	51.86 (2.21)
QI (kg/m^2^)	11.66 ± 1.27	11.90 ± 1.18	0.29	11.78 ± 1.22	11.77 ± 1.25	0.95	12.01 (1.20)
Baby head girth (cm)	33.23 ± 1.37	33.69 ± 1.21	0.05	33.39 ± 1.38	33.51 ± 1.25	0.62	33.69 (1.25)
Baby chest girth (cm)	32.75 ± 1.70	32.94 ± 1.73	0.55	32.82 ± 1.65	32.86 ± 1.79	0.91	33.07 (1.64)
1st min Apgar score	8.59 ± 1.28	8.32 ± 0.76	0.16	8.55 ± 1.34	8.37 ± 0.71	0.35	8.35 (0.75)
5th min Apgar score	9.92 ± 1.13	9.68 ± 0.47	0.15	9.91 ± 1.16	9.70 ± 0.46	0.19	9.73 (0.45)
Baby bilirubin (mg/dL)	8.80 ± 2.61	7.93 ± 2.31	0.07	8.62 ± 2.64	8.09 ± 2.33	0.27	7.96 (2.30)
Parity (%)							
1^st^	53	54	0.95^c^	58	50	0.63^c^	54 (64)
2^nd^	32	30		27	35		31 (37)
≥ 3^rd^	15	16		15	15		15 (18)

a The 17 PCDD/F and 12 dioxin-like PCB congeners with the WHO toxic equivalent factors were measured in the placenta.

b Mann-Whitney *U*-test adjusted for mother’s age for all the characteristics examined.

c Chi-square test *p*-value.

**Table 2:** Hormone concentrations by median of dioxins/PCB TEq (pg/g lipid, mean ± SD) level in the female and male babies.

	Female				Male			
Hormone	Low (< 15.1; *n* = 31)	High (≥ 15.1; *n* = 31)	*p*-Value^a^	Total	Low (< 15.2; *n* = 28)	High (≥ 15.2; *n* = 29)	*p*-Value^a^	Total
T_3_ (nmol/L)	0.79 ± 0.18	0.91 ± 0.28	0.05#	0.85 ± 0.24	0.96 ± 0.32	0.88 ± 0.24	0.34	0.92 ± 0.28
T_4_ (nmol/L)	105.8 ± 22.7	115.7 ± 19.6	0.07#	110.8 ± 21.7	112.9 ± 28.8	116.5 ± 21.3	0.59	114.8 ± 25.0
T_3_ uptake (%)	30.5 ± 4.36	28.9 ± 4.08	0.15	29.7 ± 4.26	28.1 ± 5.22	28.0 ± 3.44	0.94	28.1 ± 4.37
FT_4_ (pmol/L)	10.7 ± 3.2	10.8 ± 2.3	0.85	10.7 ± 2.8	10.4 ± 2.4	9.93 ± 2.7	0.44	10.2 ± 2.6
TSH (mU/L)	9.23 ± 5.7	6.86 ± 5.3	0.10#	8.03 ± 5.6	8.46 ± 6.5	6.99 ± 5.5	0.38	7.67 ± 6.0
TBG (mg/L)	21.9 ± 3.95	24.6 ± 4.82	0.03*	23.2 ± 4.57	25.0 ± 7.21	25.1 ± 4.47	0.94	25.0 ± 5.86
IGF-1 (ng/dL)	85.2 ± 38.6	99.1 ± 36.0	0.15	95.2 ± 37.7	82.3 ± 35.3	86.0 ± 36.6	0.70	84.3 ± 34.7
BP3 (ng/dL)	1.23 ± 0.44	1.64 ± 0.84	0.02*	1.43 ± 0.70	1.54 ± 0.86	1.38 ± 0.87	0.51	1.46 ± 0.86

a Student *t*-test or Mann-Whitney *U*-test when data distribution was significantly beyond the normal distribution range:

* *p* < 0.05,

# *p* < 0.1.

**Table 3:** Correlation^a^ between thyroid hormone concentrations in cord serum and birth-related indices.

Hormones	Maternal age (years)	Gestational age (weeks)	Placenta weight (g)	Birth weight (g)	Birth length (cm)	Baby head girth (cm)	QI (kg/m^2^)	1st min Apgar score	5th min Apgar score	Baby bilirubin (mg/dL)
Maternal age	1	−0.271**	−0.004	−0.018	0.040	0.023	−0.103	−0.191*	−0.025*	−0.054
Gestational age	−0.271**	1	0.099	0.308***	0.136	0.169#	0.355***	0.210*	0.208*	−0.015
T_3_ (nmol/L)	−0.069	0.218	−0.157	0.046	−0.006	0.044	0.105	−0.036	−0.042	0.004
T_4_ (nmol/L)	−0.105	0.137	0.020	−0.029	−0.120	−0.075	0.129	0.086	0.044	−0.070
TSH (μU/L)	−0.216	0.148	0.288**	0.155#	−0.039	0.055	0.205*	0.054	0.066	0.018
T_3_ uptake (%)	0.025	−0.244**	0.178#	0.022	0.074	0.106	−0.084	−0.049	−0.032	−0.030
FT_4_ (pmol/L)	−0.042	−0.005	0.080	0.009	−0.140	−0.055	0.171#	0.042	0.115	−0.085
TBG (mg/L)	−0.063	0.213*	0.051	0.009	−0.107	0.001	0.154	0.120	0.085	−0.062
IGF-1 (nmol/L)	0.069	−0.164#	0.279**	0.309***	0.112	0.321***	0.267**	−0.139	−0.062	−0.169#
BP3 (nmol/L)	−0.086	0.023	0.237*	0.218*	0.050	0.300**	0.234*	−0.078	−0.015	−0.119
FT_4_ × TSH	−0.224*	0.148	0.237*	0.117	−0.095	0.016	0.226*	0.081	0.154	−0.039
T_4_ × TSH	−0.255**	0.168#	0.281**	0.118	−0.089	0.002	0.229**	0.085	0.097	0.006
T_4_:TBG	−0.031	−0.007	0.004	−0.064	−0.088	−0.125	−0.009	0.008	−0.044	0.015
T_3_ uptake:T_3_	0.059	−0.248**	0.177#	−0.011	0.066	0.027	−0.116	0.011	0.040	−0.008
FT_4_:T_3_	0.023	−0.213*	0.174#	−0.054	−0.091	−0.044	0.001	−0.012	0.031	−0.079

a Spearman correlation:

*** *p* < 0.001,

** *p* < 0.01,

* *p* < 0.05,

# *p* < 0.1.

**Table 4:** Correlation^a^ between PCDD/Fs and PCB levels in toxic equivalence and thyroid hormone concentrations in cord serum by infant’s sex.

Hormone	Infant sex	Mother’s age	PCDDs	PCDFs	Non-*ortho* PCBs	Mono-*ortho* PCBs	PCB-138, -153, -180
T_3_ (nmol/L)	F	0.097	0.214	0.300*	0.216	0.227#	0.074
	M	−0.220	−0.090	−0.039	−0.008	0.070	0.035
T_4_ (nmol/L)	F	0.066	0.191	0.253#	0.189	0.191	0.282#
	M	−0.236#	0.116	0.231#	−0.137	0.172	0.183
TSH (μU/L)	F	−0.223#	−0.265*	−0.233#	−0.320*	−0.264*	−0.154
	M	−0.225	−0.214	0.015	−0.130	−0.239#	−0.078
T_3_ uptake (%)	F	−0.059	−0.177	−0.172	0.074	0.024	0.019
	M	0.057	0.007	−0.048	−0.003	−0.237#	−0.179
FT_4_ (pmol/L)	F	0.062	−0.004	−0.017	0.058	0.022	0.247#
	M	−0.172	−0.182	0.058	−0.302*	−0.178	−0.033
TBG (mg/L)	F	0.121	0.319*	0.368**	0.118	0.189	0.136
	M	−0.176	−0.039	0.118	−0.141	0.107	0.054
IGF-1 (nmol/L)	F	0.175	0.073	0.053	−0.035	−0.110	−0.196
	M	−0.084	−0.045	−0.006	−0.179	−0.061	−0.018
BP3 (nmol/L)	F	0.161	0.185	0.254#	−0.097	−0.061	−0.215
	M	−0.329*	−0.190	−0.141	−0.204	−0.253	−0.208
FT_4_ × TSH	F	−0.239#	−0.218	−0.187	−0.294*	−0.243#	−0.210
	M	−0.249	−0.210	0.047	−0.291	−0.276	−0.035
T_4_:TBG	F	−0.067	−0.305*	−0.270#	0.046	−0.068	0.082
	M	−0.002	0.082	0.049	0.057	−0.002	0.110
T_3_ uptake:T_3_	F	−0.073	−0.224#	−0.282*	−0.144	−0.152	< 0.001
	M	0.146	0.021	−0.023	0.014	−0.156	−0.151
FT_4_:T_3_	F	−0.063	−0.188	−0.242#	−0.134	−0.162	0.131
	M	0.088	−0.059	0.023	−0.188	−0.176	−0.040

a Spearman correlation:

** *p* < 0.01,

* *p* < 0.05,

# *p* < 0.1.

**Table 5:** Correlation coefficients^a^ between PCDD/F and PCB levels and thyroid hormone concentrations in cord serum by stepwise multivariate linear regression.

Hormone	Mother’s age	PCDD/Fs	Non-*ortho* PCBs
T_4_	−0.274**	0.202#	—
TBG	−0.201#	0.286*	—
FT_4_	—	—	−0.277**
FT_4_ × TSH	−0.218*	—	−0.246*

a The hormones levels were log-transformed for normality to use the parametric method.

** *p* < 0.01,

* *p* < 0.05,

# *p* < 0.1.
